# Supplementary material for: Causal Relationship between Aging and Anorexia Nervosa: A White-Matter-Microstructure-Mediated Mendelian Randomization Analysis
Source: Biomedicines. 2024 Aug 16;12(8):1874. doi: 10.3390/biomedicines12081874 (PMC11351342; doi:10.3390/biomedicines12081874)
Supplement: Supplementary file 1 [file biomedicines-12-01874-s001.zip › Supplementary Figure/Figure S4/Singe SNP plot of MR analysis for TL to ubm-b-1647.pdf]

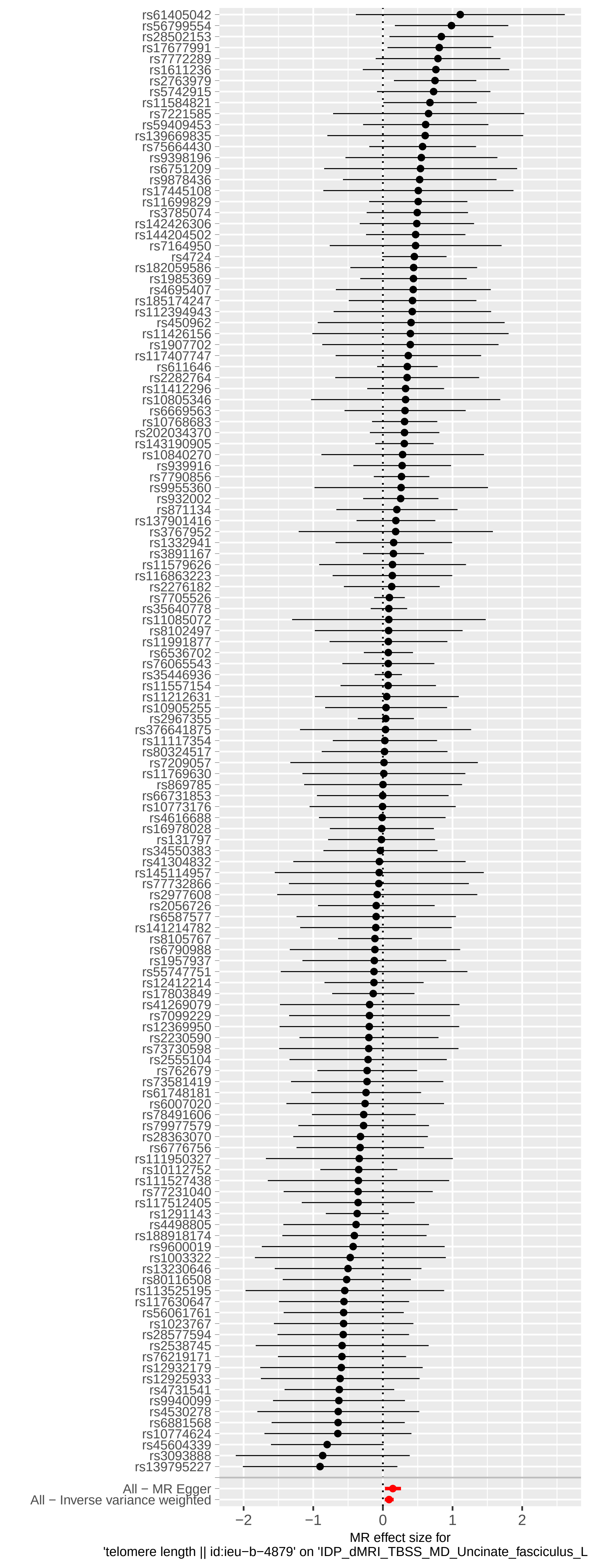

rs61405042
rs56799554
rs28502153
rs17677991
rs7772289
rs1611236
rs2763979
rs5742915
rs11584821
rs7221585
rs59409453
rs139669835
rs75664430
rs9398196
rs6751209
rs9878436
rs17445108
rs11699829
rs3785074
rs142426306
rs144204502
rs7164950
rs4724
rs182059586
rs1985369
rs4695407
rs185174247
rs112394943
rs450962
rs11426156
rs1907702
rs117407747
rs611646
rs2282764
rs11412296
rs10805346
rs6669563
rs10768683
rs202034370
rs143190905
rs10840270
rs939916
rs7790856
rs9955360
rs932002
rs871134
rs137901416
rs3767952
rs1332941
rs3891167
rs11579626
rs116863223
rs2276182
rs7705526
rs35640778
rs11085072
rs8102497
rs11991877
rs6536702
rs76065543
rs35446936
rs11557154
rs11212631
rs10905255
rs2967355
rs376641875
rs11117354
rs80324517
rs7209057
rs11769630
rs869785
rs66731853
rs10773176
rs4616688
rs16978028
rs131797
rs34550383
rs41304832
rs145114957
rs77732866
rs2977608
rs2056726
rs6587577
rs141214782
rs8105767
rs6790988
rs1957937
rs55747751
rs12412214
rs17803849
rs41269079
rs7099229
rs12369950
rs2230590
rs73730598
rs2555104
rs762679
rs73581419
rs61748181
rs6007020
rs78491606
rs79977579
rs28363070
rs6776756
rs111950327
rs10112752
rs111527438
rs77231040
rs117512405
rs1291143
rs4498805
rs188918174
rs9600019
rs1003322
rs13230646
rs80116508
rs113525195
rs117630647
rs56061761
rs1023767
rs28577594
rs2538745
rs76219171
rs12932179
rs12925933
rs4731541
rs9940099
rs4530278
rs6881568
rs10774624
rs45604339
rs3093888
rs139795227
All − MR Egger
All − Inverse variance weighted
−2
−1
0
1
2
MR effect size for
'telomere length || id:ieu−b−4879' on 'IDP_dMRI_TBSS_MD_Uncinate_fasciculus_L
